# Closed head experimental traumatic brain injury increases size and bone volume of callus in mice with concomitant tibial fracture

**DOI:** 10.1038/srep34491

**Published:** 2016-09-29

**Authors:** Rhys D Brady, Brian L Grills, Jarrod E Church, Nicole C Walsh, Aaron C McDonald, Denes V Agoston, Mujun Sun, Terence J O’Brien, Sandy R Shultz, Stuart J McDonald

**Affiliations:** 1 Department of Physiology, Anatomy and Microbiology, La Trobe University, VIC, 3086, Australia; 2 Murdoch Childrens Research Institute, Royal Children’s Hospital, Flemington Road, Parkville, VIC 3052, Australia; 3 Department of Anatomy, Physiology, and Genetics. Uniformed Services University of the Health Sciences, Bethesda, MD, USA; 4 Department of Medicine, The Royal Melbourne Hospital, The University of Melbourne, VIC, 3010, Australia

## Abstract

Concomitant traumatic brain injury (TBI) and long bone fracture are commonly observed in multitrauma and polytrauma. Despite clinical observations of enhanced bone healing in patients with TBI, the relationship between TBI and fracture healing remains poorly understood, with clinical data limited by the presence of several confounding variables. Here we developed a novel trauma model featuring closed-skull weight-drop TBI and concomitant tibial fracture in order to investigate the effect of TBI on fracture healing. Male mice were assigned into Fracture + Sham TBI (FX) or Fracture + TBI (MULTI) groups and sacrificed at 21 and 35 days post-injury for analysis of healing fractures by micro computed tomography (μCT) and histomorphometry. μCT analysis revealed calluses from MULTI mice had a greater bone and total tissue volume, and displayed higher mean polar moment of inertia when compared to calluses from FX mice at 21 days post-injury. Histomorphometric results demonstrated an increased amount of trabecular bone in MULTI calluses at 21 days post-injury. These findings indicate that closed head TBI results in calluses that are larger in size and have an increased bone volume, which is consistent with the notion that TBI induces the formation of a more robust callus.

Concomitant traumatic brain injury (TBI) and long bone fracture are present in many multitrauma and polytrauma patients as a consequence of high energy impacts that result from motor vehicle collisions, falls and warzone injuries^1^,^2^,^3^. Long bone fracture healing involves a biological sequence of four overlapping phases; an inflammatory phase, which features migration of inflammatory cells and the release of chemokines and cytokines that initiate the healing response, formation of a fibrocartilaginous soft callus to bridge the fractured bone ends, replacement of soft callus with bony hard callus in order to restore mechanical stability, and a remodelling phase that restores bone to its pre-injured state^4^.

TBI is a complex disease induced by external mechanical forces to the brain, and the consequent biological response results from a combination of primary injuries that occur at the moment of impact, and the subsequent complex cascade of secondary injury pathways that may develop over the hours, days, or weeks that follow^5^,^6^. TBI is recognised to induce both central and systemic changes^7^, a number of which have potential to affect bone and bone fracture healing. We previously demonstrated that experimental TBI in the absence of fracture caused significant systemic bone loss with reductions in cortical and trabecular bone volume in rats^8^. Clinical studies have also reported lower bone mineral density and an elevated risk of fracture in brain-injured patients^9^,^10^,^11^,^12^. Paradoxically, TBI has been associated with stimulation of osteogenesis, with heterotopic ossification (i.e. the formation of bone in soft tissues) and enhanced callus formation described in TBI patients for several decades^13^,^14^,^15^,^16^,^17^. Despite these associations the relationship between TBI and fracture healing remains poorly understood. Clinical data is limited by the presence of several confounding variables and results from initial rodent studies are mixed. In addition, nearly all previous rodent studies have administered TBI using the controlled cortical impact (CCI) model that requires craniotomy^18^,^19^,^20^,^21^,^22^. Given that previous studies have demonstrated enhanced osteogenesis in distant skeletal sites following bone injury, possibly through release of osteogenic humoral factors via a process known as ‘systemic acceleration’^23^, the craniotomy performed to administer the CCI most likely represents a confounding variable.

Despite the fact that closed-skull brain injury is the most common form of TBI in humans^24^,^25^, the bone healing response of rodents exposed to closed-skull TBI (i.e. without craniotomy) remains unclear. Accordingly, in order to further explore the effect of closed-skull TBI on fracture healing, we developed a novel combined trauma mouse model that involved a weight-drop TBI and concomitant tibial fracture.

## Materials and Methods

### Mice

C57Bl/6 male mice were obtained from the Australian Animal Resource Centre (ARC, Western Australia) for use in this study. Mice were 12 weeks of age at the time of injury, were housed individually under a 12-hour light/dark cycle, and were given access to food and water *ad libitum* for the duration of the experiment. All procedures were approved by The Florey Institute of Neuroscience and Mental Health Animal Ethics Committee (#14-006-UM), were within the guidelines of the Australian code of practice for the care and use of animals for scientific purposes by the Australian National Health and Medical Research Council, and in compliance with the ARRIVE guidelines for how to report animal experiments.

### Experimental Groups

Mice were randomly assigned to either Fracture + Sham (FX) or Fracture + TBI (MULTI) injury groups. Thirty-three mice were used for this experiment including 4 mice which died following TBI. The FX group consisted of 14 mice and the MULTI group consisted of 15 mice. Mice were either killed at 21 days post-injury (FX, n = 6; MULTI, n = 9) or at 35 days post-injury (FX, n = 8; MULTI, n = 6). Fractures were analysed via μCT and histological analysis was performed on 22 of 29 fracture samples, with 7 specimens (4 MULTI, 3 FX) omitted due to complications during processing.

### Tibial Fracture

**Figure 1:**
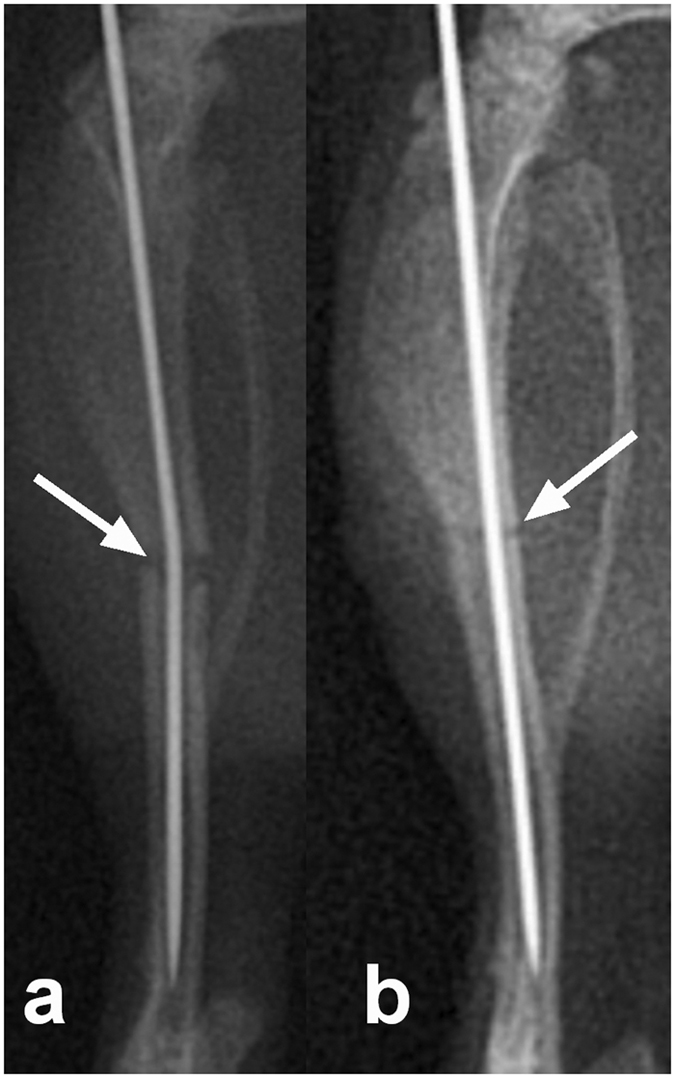
Tibial fracture was generated in the mid-diaphysis (arrow) above the fibular junction following intramedullary pinning (**a**), and following confirmation of the tranverse, non-comminuted fracture the intramedullary rod was replaced with a new, larger rod (**b**).

Before the assigned TBI injury was given, mice received a closed tibial fracture stabilised by intramedullary fixation as previously described^26^. Mice were anaesthetised using a mixture of oxygen and 4% isoflurane and anesthesia was maintained using a mixture of oxygen and 2% isoflurane. A small incision was then made inferior of the right knee, an entry point into the medullary canal of the tibia was made using a 26-G needle, and an intramedullary rod (000 insect pin, 0.25 mm diameter) was inserted inside the medullary canal. A fracture was then generated in the tibial midshaft using three-point bending tweezers, and the fracture was visualised using radiography for confirmation of a transverse, non-comminuted fracture supported by the intramedullary rod (Fig. 1a). Following the fracture, the initial intramedullary rod was replaced with a new rod (00 insect pin, 0.30 mm diameter) which remained *in situ* for the remainder of the study to ensure alignment of fractured-ends (Fig. 1b). The incision was then sutured. Sham-injury for the fracture procedure consisted of the incision and suturing, but no fracture was generated.

### Closed-skull weight-drop model of TBI

Weight-drop TBI and associated sham-injury procedures were based on previously described standard protocols^27^,^28^. Briefly, the weight-drop device consisted of a guided- and weighted-rod (215 g) with a blunt silicone-covered impact tip (4 mm diameter). A longitudinal incision was made along the midline of the scalp and following tibial fracture the mouse was stabilised on the injury device platform. The weighted rod was released from a height of 2 cm, and the impact tip made contact between the sagittal and coronal suture. The TBI sham-injury procedure was identical to that described for the TBI procedure, except the weighted rod was not released. All mice received 0.05 mg/kg of buprenorphine analgesic subcutaneously post-injuries.

### Acute assessment of injury severity

Apnea, unconsciousness and self-righting reflex times shown in Table 1, were monitored in all mice immediately after injury and were indicators of acute injury severity. Apnea was the time from injury to spontaneous breathing. Loss of consciousness was the time from injury to a hind-limb withdrawal response to a toe pinch. Self-righting reflex was the time from injury to the return of an upright position.

### μCT

Fractured tibia were fixed overnight in 4% paraformaldehyde in 0.1 M sodium cacodylate buffer (pH 7.4) and stored at 4 °C in 0.1 M cacodylate buffer containing 10% sucrose (pH 7.4)^29^. Images were acquired using a Skyscan 1076 scanner (Bruker-microCT) at 9 μm voxel resolution, 0.5 mm aluminium filter, 48 kV voltage, 100 μA current, 2400 ms exposure, rotation 0.5° across 180°, frame averaging of 1. Images were reconstructed using NRecon (version 1.6.3.1) and the following parameters: CS to image conversion, 0.0–0.11; ring artefact, 6; pixel defect mask, 5%; and beam hardening correction, 35%. Following reconstruction, the region of interest (ROI) for each bone was determined using CTAN (version 1.11.8.0, Bruker MicroCT) as being a 2 mm region longitudinally centred on the callus (i.e. 1 mm either side of the fracture line of the callus); the border of the callus was deleniated using the “shrink-wrap” function. Thresholds used for quantification of structural parameters were determined using the automatic “otsu” algorithm within CTAn, and visual inspection of images and qualitative comparison with histological sections. Once determined, a threshold of 41 was used for structural analysis of calluses at both 21 and 35 days post-fracture. 2D and 3D data were generated for all analyses and 3D models were generated using the “marching cubes” algorithm from thresholded data (in CTAn). Following imaging, calluses were prepared for histology.

### Histological processing, staining, and histomorphometry

Scanned tibial fractures were processed to plastic, sectioned and stained. Briefly, tibial fractures were dehydrated using a graded series of ethanols and infiltrated and embedded in LR White resin (London Resin Company limited, Reading, England). Samples were polymerised in LR White resin at 60 °C for 24 h. Five micron thick longitudinal sections were cut at the midpoint of undecalcified callus on a Leica RM 2155 Rotary Microtome (Leica, Wetzlar, Germany) with a tungsten carbide blade. Sections were stained using Safranin O and Fast green to examine bone and cartilage content. Additional sections were also stained for the presence of tartrate-resistant acid phosphatase (TRAP; commonly used as a cytochemical marker of osteoclasts). The total area of the callus stained positive for TRAP activity was divided by the total callus area to yield the percentage of the callus occupied by TRAP activity as a surrogate marker for osteoclast area. Sections were photographed on a Leica DMBRE microscope (magnification 25x for Safranin O and Fast green stained sections and 50x for TRAP stained sections) before being assessed both qualitatively and quantitatively using Leica Qwin software.

### Statistical analysis

All data was analysed with GraphPad prism 6 (GraphPad software, Inc.) using Mann-Whitney-U tests with significance defined as p < 0.05.

## Results

### μCT

**Figure 2:**
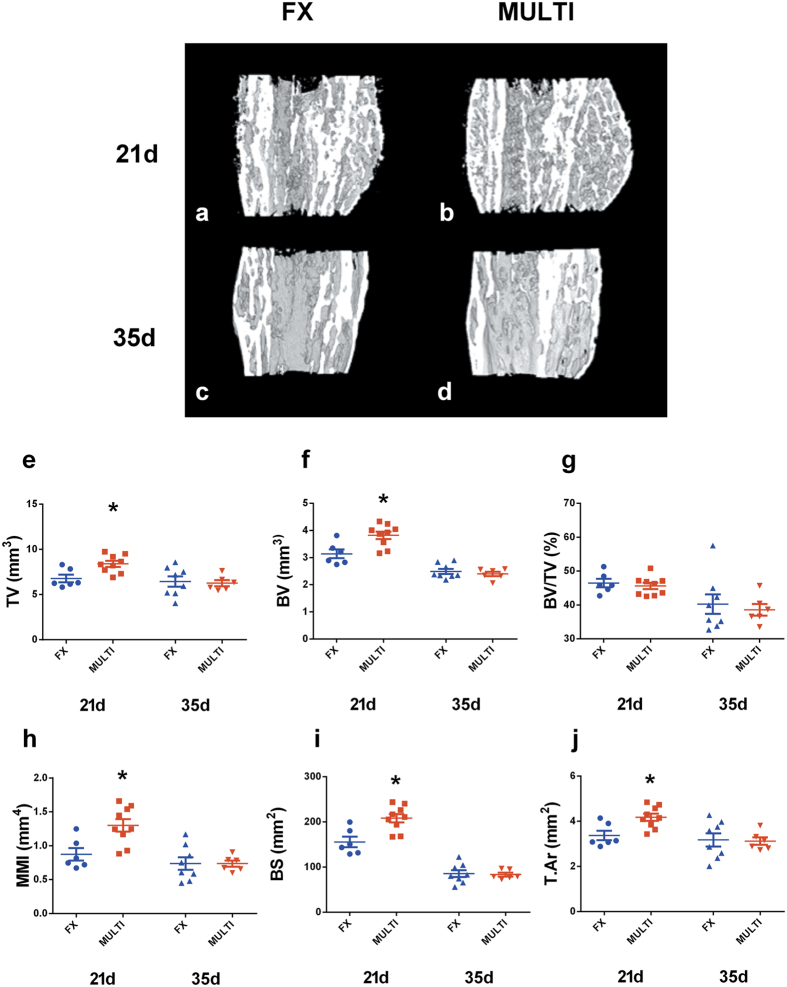
Longitudinal midpoint views of representative μCT reconstructions of hemi calluses (**a**–**d**). μCT analysis of fracture calluses shows MULTI calluses (n = 9) had increased total volume (**e**; TV), bone volume (**f**; BV), mean polar moment of inertia (**h**; MMI), bone surface (**i**; BS) and mean tissue area (**j**; T.Ar) at 21 days post-injury compared to FX calluses (n = 6; *p < 0.05). However, there was no difference in bone volume fraction (**g**; BV/TV) between MULTI and FX calluses. No differences were found between 35 day fracture calluses from FX (n = 8) and MULTI (n = 6) mice (**e**–**j**). Bars are means ± SEM.

Representative μCT reconstructions of longitudinal midpoint hemi-calluses are shown in Fig. 2(a–d). μCT assessment revealed that at 21 days post-fracture all calluses from both injury groups had reached union. Analysis of calluses at 21 days post-injury (Fig. 2e–j) revealed MULTI calluses had significantly greater total volume (e; 21%. p < 0.05), bone volume (f; 19%. p < 0.05), and mean polar moment of inertia (h; 40%. p < 0.05), bone surface (i; 29%. p < 0.05) and mean tissue area (j; T.Ar. 21%. p < 0.05) when compared to calluses from FX mice, however, no difference in bone volume fraction (g; BV/TV) between MULTI and FX calluses was observed. No differences between FX and MULTI calluses were observed at 35 days post injury in any measured parameter (e–j).

### Histology and histomorphometric analysis

**Figure 3:**
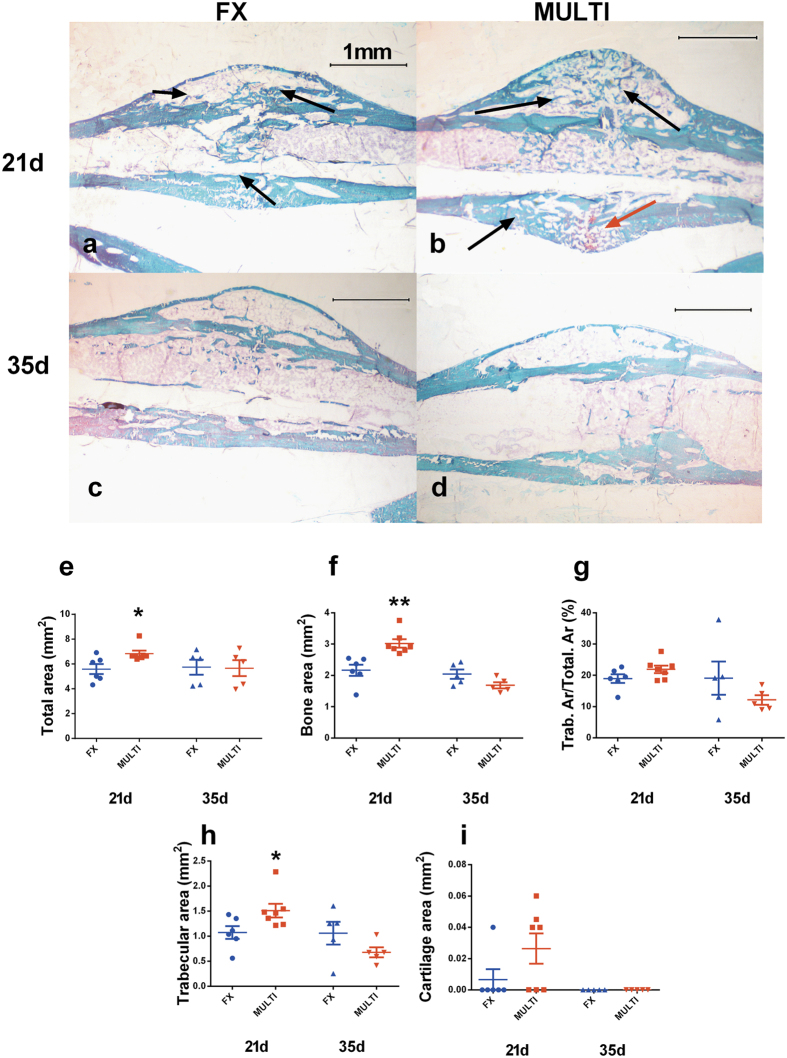
Representative histological sections of undecalcified calluses from FX and MULTI mice at 21 and 35 days post-injury (**a**–**d**, magnification 25x). Histological assessment at 21 days post-injury shows an increased amount of newly formed mineralized trabecular bone (stained green; black arrows) and presence of non-mineralized cartilage (stained red; red arrow) in MULTI calluses (**b**; n = 6) compared to FX calluses (**a**; n = 6), however at 35 days there are no obvious differences between MULTI (**d**; n = 5) and FX calluses (**c**; n = 5). Histomorphometric analysis of fracture calluses shows MULTI calluses had increased total area (**e**; *p < 0.05), bone area (**f**; **p < 0.01) and area of newly formed trabecular bone (**h**; *p < 0.05), at 21 days post-injury when compared to calluses from FX mice. However, no difference in newly formed bone area fraction (**g**; Trab.Ar/Total.Ar), or cartilage area (**i**) was detected between MULTI and FX calluses. No differences were observed in day 35 fracture calluses between FX and MULTI mice (**e**–**i**). Bars are means ± SEM.

Representative histological sections are shown in Fig. 3(a–d). Qualitative histological assessment of calluses at 21 days post-injury showed an obvious increase in trabecular bone in MULTI calluses (b) compared to FX calluses (a). By 35 days there were no obvious qualitative differences in the histological appearance between MULTI (d) and FX calluses (c) in concordance with μCT. Histomorphometric analysis reflected the qualitative histological assessment of calluses and complemented μCT analysis. At 21 days post-injury, compared to FX calluses, MULTI calluses had increased total area (e; 20%. p < 0.05), bone area (f; 33%. p < 0.01) and area of newly formed trabecular bone (h; 30%. p < 0.05, however, no difference in newly formed bone area fraction (g; Trab.Ar/Total.Ar), or cartilage area (i) was detected between MULTI and FX calluses. At 35 days post-injury no differences were found between FX and MULTI calluses in any measured parameter (e–i).

**Figure 4:**
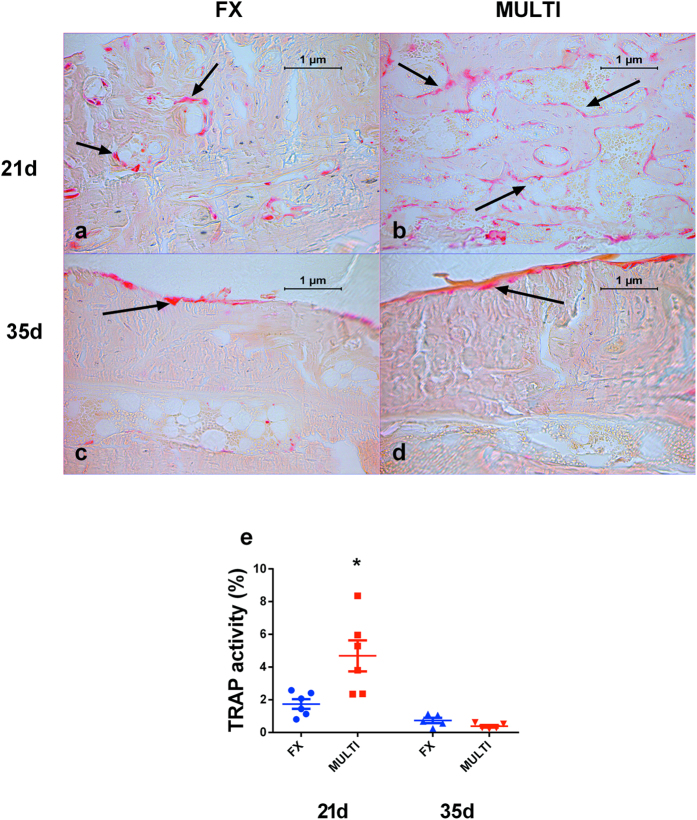
Representative histological sections of un-decalcified calluses from FX and MULTI mice at 21 and 35 days post-injury (**a**–**d**; magnification 200x). Histological assessment at 21 days post-injury shows an increased amount of TRAP activity stained red in MULTI calluses (**b**; n = 6) compared to FX calluses (**a**; n = 6), however at 35 days there are no obvious differences between MULTI (**d**; n = 5) and FX calluses (**c**; n = 5). Analysis of fracture calluses shows MULTI calluses had increased percentage of TRAP activity (**e**; *p < 0.05) compared to calluses from FX mice. However at 35 days post-fracture there were no differences between the groups (**e**). Bars are means ± SEM.

Representative TRAP stained histological sections are shown in Fig. 4(a–d). Qualitative assessment of calluses shows an increase in TRAP activity in MULTI calluses (b) compared to FX calluses (a) at 21 days post-injury. Quantitative analysis of 21 day calluses revealed a significant increase in the TRAP activity/total callus area indicating a greater density of osteoclast-lineage cells in calluses from MULTI mice compared to calluses from fracture-only mice (e; p < 0.05). No difference in the extent of area stained for TRAP activity/total callus area was found at 35 days post-fracture (c–e).

## Discussion

Few studies have investigated the effect of closed-skull TBI on fracture healing, therefore here we have developed a novel mouse model that involved a closed-skull weight-drop TBI and concomitant tibial fracture. In this study we have shown that in mice subjected to closed-skull TBI, fracture calluses at day 21 were larger, had a greater bone volume, and displayed a higher mean polar moment of inertia when compared to calluses from fracture-only mice. By day 35 the difference in fracture callus was not evident. While several previous studies have reported that TBI increased fracture callus size and extent of mineralization, these studies all featured an open-skull TBI model using craniotomy^18^,^19^,^20^,^21^,^22^, which represents a potentially confounding factor. This study, to the best of our knowledge, is the first to document that a closed-skull model of TBI enhanced formation of fracture callus in long bones.

Our findings are similar to those previously described in rodents given open-skull CCI, with several studies similarly reporting greater callus bone and total tissue volume between 2–4 weeks post-TBI^18^,^19^,^20^. In addition, the increased mean polar moment of inertia (a quantity used to predict an object's ability to resist torsion) observed in calluses from mice with TBI indicated that closed-skull TBI may have resulted in superior mechanical integrity of the fracture site. Taken together, these findings suggest that closed-skull TBI enhanced callus formation rate and/or reduced bone resorption and thereby likely resulting in a decreased risk of re-fracture at 21 days post-injury. Importantly, results from this study suggest that the heightened callus formation described in previous studies is unlikely to be primarily due to the craniotomy procedure, although in the absence of appropriate controls (i.e. craniotomy + fracture), the contribution of ‘systemic acceleration’ in these studies cannot be discounted^23^.

The results of this study provide insight into the nature of callus modelling/remodelling following TBI. Although there were no significant differences in cartilage volumes between groups, four of the seven calluses from brain-injured mice contained non-mineralized cartilage, compared with one of the seven calluses from fracture-only mice. This finding may suggest that calluses from mice with TBI featured a relatively higher degree of cartilage formation and endochondral ossification, however further investigations are required to fully characterise the nature of callus modelling/remodelling in animals with TBI in the early stages post-injury. Structural differences in calluses from brain-injured and fracture-only mice did not persist to 35 days post-injury^21^,^22^. This result, combined with our finding of an increased percentage of TRAP-activity in calluses from brain-injured mice at 21 days post-injury, is likely to indicate that calluses from mice with TBI underwent significant resorption between days 21 and 35 in order to remodel to the size of their fracture-only counterparts at 35 days post-fracture.

We observed that calluses from multiply-injured mice were larger and had a greater bone volume at 21 days post-fracture compared to those from fracture only mice. These results conflict with the findings of Boes and colleagues, who reported that calluses from mice with closed-skull injury were reduced in size^30^. This discrepancy in findings may be attributed to differences in the nature of the weight-drop brain injury, with the model of Boes *et al*. inducing a highly diffuse injury and the model of the current study causing a mixed focal/diffuse injury pattern^27^,^30^. Acute measures of injury severity in this study indicate that our TBI model was of mild-moderate severity, given that previous studies have speculated that callus size may correlate with TBI severity^31^, it is possible that an increased severity of experimental TBI may result in callus size remaining larger at time-points beyond 21 days post-injury. Additional studies using a variety of injury parameters, including differences in injury location, severity and mechanism, are required to provide further insights into how particular TBIs may impact callus formation.

Though several studies have reported enhanced callus volumes post-TBI^18^,^19^,^20^,^21^,^22^, the nature of callus formation following TBI is not well established. Several researchers have speculated that the enhanced callus volumes post-TBI may represent a form of heterotopic ossification about the fracture site^16^,^17^,^31^,^32^,^33^. We observed no gross morphological differences in healing calluses between multitrauma and fracture-only mice using high resolution μCT and histology, however the contribution of heterotopic ossification to an enhanced callus formation response cannot be ruled out. Notably, a recent study has provided insight into the possible pathogenesis of neurological heterotopic ossification, finding bone formation in the hamstring of mice with combined spinal cord injury and muscle injury, but not following either injury in isolation^34^. The authors also found depletion of phagocytic macrophages reduced heterotopic ossification volume by 90%, which suggests that localised inflammation in combination with neurological injury may drive this abnormal bone formation. Accordingly, in our study it is possible that the inflammatory response to tibial fracture, in combination with TBI, may have provided the osteogenic conditions necessary to enhance bone formation about the fracture site.

There are several potential mechanisms through which the central and systemic changes occurring post-TBI may influence bone healing^13^,^14^,^15^,^16^,^17^. Growing evidence suggests a significant role for the central nervous system in regulating bone homeostasis^35^,^36^,^37^,^38^,^39^,^40^, it is possible an injury-induced disturbance of these neural pathways may alter the bone modelling/remodelling response during fracture healing. Furthermore, several *in vitro* studies have demonstrated that serum from brain-injured rats increased the proliferation of mesenchymal stem cells, and that serum and CSF from TBI patients increased osteoblastic proliferation^30^,^41^,^42^. These results have led to speculation that the enhanced callus formation observed following TBI may be due to an unknown humoral mechanism^16^,^17^. Further studies are required to identify the possible humoral factor/s and the extracellular and intracellular signalling pathways responsible for the enhanced callus formation following TBI.

Though we have provided evidence for the first time that closed head TBI results in increased callus formation, this study has limitation. We have analysed two defined time-points following fracture healing and clearly show that TBI enhanced callus size at 21 days post fracture. These are static analyses and provide a snapshot of cell activity at that given time. Further studies using *in vivo* μCT or serial x-ray analyses to assess changes in the fracture sites/fracture callus overtime, will provide insight into the timing of callus formation and fracture union. In addition, greater understanding of the cellular mechanism which resulted in enlarged callus formation will be possible with future studies utilising *in vivo* fluorochrome labelling to definitively determine bone formation rates, and static bone histomorphometry to determine osteoclast/osteoblast numbers. Furthermore, analysis of serum markers for bone and cartilage formation and resorption could provide additional insight in to the timing and extent of these cellular processes during callus formation and remodelling. However, recent studies by our laboratory have shown that TBI leads to reduction in bone volume in un-fractured limbs^8^, therefore analysis of serum bone turnover markers may not provide accurate indication of fracture-specific remodelling and histomorphometric analyses combined with radiographic analyses may be a more informative measure. Finally, it is important to recognise that the initial fracture healing cascade may have been influenced by any TBI-induced reductions in animal mobility in the early stages post-injury. Nonetheless, a recent study which characterised the weight-drop injury model used in this study reported no significant deficits on a neurological severity scale (of which motor function was a key outcome) by 24 hours post-injury^27^. Accordingly, the authors believe mobility was not reduced in mice with TBI, and therefore did not influence the findings of the current study. However, future investigations should monitor mobility/activity levels of mice in the early stages post-TBI in order to rule out a reduction in activity levels as a confounding factor.

## Conclusion

The findings presented in this study indicate that long bone fracture in closed head TBI results in calluses that are larger in size and have an increased bone volume compared to calluses from fracture-only mice. This finding is consistent with the notion that TBI leads to a heightened callus formation response. Although future studies are required to elucidate the mechanisms behind this phenomenon, these findings improve our understanding of the effect of TBI on bone regeneration and allow us to better understand the complex nature of combined traumatic injuries.

## Additional Information

**How to cite this article**: Brady, R. D. *et al*. Closed head experimental traumatic brain injury increases size and bone volume of callus in mice with concomitant tibial fracture. *Sci. Rep.* **6**, 34491; doi: 10.1038/srep34491 (2016).

## Figures and Tables

**Table 1:** Acute injury severity measures.

	Apnea	Hindlimb	Self-righting
FX	0	46.6 ± 15.6	68 ± 14.7
MULTI	19.7 ± 3.9^a^	179.9 ± 43.4^b^	204.4 ± 41.9^b^

The MULTI group experienced significantly longer apnea, hind-limb withdrawal, and self-righting reflex times (seconds) compared to the FX group. ^a^Greater than FX, p < 0.0001. ^b^Greater than FX, p < 0.01.

## References

[b1] Menetrez J. S. Inpatient multitrauma rehabilitation in a U.S. military hospital. Phys. Med. Rehabil. Clin. N. Am. 13, 67–83 (2002). 11878084 10.1016/s1047-9651(03)00072-x

[b2] Al-Thani H. . Workplace-related traumatic injuries: insights from a rapidly developing Middle Eastern country. J. Environ. Public Health 2014, 430832 (2014). 10.1155/2014/430832 PMC3964690 24734049

[b3] McDonald S. J., Sun M., Agoston D. V. & Shultz S. R. The effect of concomitant peripheral injury on traumatic brain injury pathobiology and outcome. J. Neuroinflammation 13, 90 (2016). 27117191 10.1186/s12974-016-0555-1 PMC4847339

[b4] Schindeler A., McDonald M. M., Bokko P. & Little D. G. Bone remodeling during fracture repair: The cellular picture. Semin. Cell Dev. Biol. 19, 459–466 (2008). 18692584 10.1016/j.semcdb.2008.07.004

[b5] Kumar A. & Loane D. J. Neuroinflammation after traumatic brain injury: opportunities for therapeutic intervention. Brain. Behav. Immun. 26, 1191–1201 (2012). 22728326 10.1016/j.bbi.2012.06.008

[b6] Xiong Y., Mahmood A. & Chopp M. Animal models of traumatic brain injury. Nat. Rev. Neurosci. 14, 128–142 (2013). 23329160 10.1038/nrn3407 PMC3951995

[b7] Masel B. E. & DeWitt D. S. Traumatic brain injury: a disease process, not an event. J. Neurotrauma 27, 1529–1540 (2010). 20504161 10.1089/neu.2010.1358

[b8] Brady R. D. . Experimental traumatic brain injury induces bone loss in rats. J. Neurotrauma (2015). 10.1089/neu.2014.3836 25686841

[b9] Beaupre G. S. & Lew H. L. Bone-density changes after stroke. Am. J. Phys. Med. Rehabil. 85, 464–472 (2006). 16628156 10.1097/01.phm.0000214275.69286.7a

[b10] Smith E., Comiskey C. & Carroll A. Prevalence of and risk factors for osteoporosis in adults with acquired brain injury. Ir. J. Med. Sci. 185, 473–481 (2016). 26787314 10.1007/s11845-016-1399-5

[b11] Smith E. M., Comiskey C. M. & Carroll A. M. A study of bone mineral density in adults with disability. Arch. Phys. Med. Rehabil. 90, 1127–1135 (2009). 19577025 10.1016/j.apmr.2008.09.578

[b12] Oppl B. . Low bone mineral density and fragility fractures in permanent vegetative state patients. J. Bone Miner. Res. 29, 1096–1100 (2014). 24470043 10.1002/jbmr.2122

[b13] Zhang L., Zhang L., Mao Z. & Tang P. Semaphoring 3A: an association between traumatic brain injury and enhanced osteogenesis. Med. Hypotheses 81, 713–714 (2013). 23932049 10.1016/j.mehy.2013.07.034

[b14] Wildburger R. . Post-traumatic hormonal disturbances: prolactin as a link between head injury and enhanced osteogenesis. J. Endocrinol. Invest. 21, 78–86 (1998). 9585380 10.1007/BF03350319

[b15] Wildburger R.. Post-traumatic changes in insulin-like growth factor type 1 and growth hormone in patients with bone fractures and traumatic brain injury. Wien Klin Wochenschr 113, 119–126 (2001). 11253737

[b16] Hofman M. . Improved fracture healing in patients with concomitant traumatic brain injury: proven or not? Mediators Inflamm. 2015, 204842 (2015). 25873754 10.1155/2015/204842 PMC4385630

[b17] Morley J., Marsh S., Drakoulakis E., Pape H. C. & Giannoudis P. V. Does traumatic brain injury result in accelerated fracture healing? Injury 36, 363–368 (2005). 15710151 10.1016/j.injury.2004.08.028

[b18] Tsitsilonis S. . The effect of traumatic brain injury on bone healing: an experimental study in a novel *in vivo* animal model. Injury 46, 661–665 (2015). 25682315 10.1016/j.injury.2015.01.044

[b19] Locher R. J. . Traumatic brain injury and bone healing: radiographic and biomechanical analyses of bone formation and stability in a combined murine trauma model. J. Musculoskelet. Neuronal Interact. 15, 309–315 (2015). 26636276 PMC5628590

[b20] Liu X. . SDF-1 promotes endochondral bone repair during fracture healing at the traumatic brain injury condition. PLoS One 8, e54077 (2013). 23349789 10.1371/journal.pone.0054077 PMC3551938

[b21] Wang L. . Effect of leptin on bone metabolism in rat model of traumatic brain injury and femoral fracture. Chin. J. Traumatol. 14, 7–13 (2011). 21276361

[b22] Wei Y., Wang L., Clark J. C., Dass C. R. & Choong P. F. Elevated leptin expression in a rat model of fracture and traumatic brain injury. J. Pharm. Pharmacol. 60, 1667–1672 (2008). 19000372 10.1211/jpp/60.12.0013

[b23] Mueller M., Schilling T., Minne H. W. & Ziegler R. A systemic acceleratory phenomenon (SAP) accompanies the regional acceleratory phenomenon (RAP) during healing of a bone defect in the rat. J. Bone Miner. Res. 6, 401–410 (1991). 1858523 10.1002/jbmr.5650060412

[b24] Masson F. . Epidemiology of severe brain injuries: a prospective population-based study. J. Trauma 51, 481–489 (2001). 11535895 10.1097/00005373-200109000-00010

[b25] Myburgh J. A. . Epidemiology and 12-month outcomes from traumatic brain injury in australia and new zealand. J. Trauma 64, 854–862 (2008). 18404048 10.1097/TA.0b013e3180340e77

[b26] Schindeler A. . Models of tibial fracture healing in normal and Nf1-deficient mice. J. Orthop. Res. 26, 1053–1060 (2008). 18383150 10.1002/jor.20628

[b27] Flierl M. A. . Mouse closed head injury model induced by a weight-drop device. Nat. Protoc. 4, 1328–1337 (2009). 19713954 10.1038/nprot.2009.148

[b28] Shultz S. R. . Tibial fracture exacerbates traumatic brain injury outcomes and neuroinflammation in a novel mouse model of multitrauma. J. Cereb. Blood Flow Metab. 35, 1339–1347 (2015). 25853909 10.1038/jcbfm.2015.56 PMC4528010

[b29] Brady R. D. . Thymosin beta4 administration enhances fracture healing in mice. J. Orthop. Res. 32, 1277–1282 (2014). 25042765 10.1002/jor.22686

[b30] Boes M. . Osteogenic effects of traumatic brain injury on experimental fracture-healing. J. Bone Joint Surg. Am. 88, 738–743 (2006). 16595463 10.2106/JBJS.D.02648

[b31] Spencer R. F. The effect of head injury on fracture healing. A quantitative assessment. J. Bone Joint Surg. Br. 69, 525–528 (1987). 3611151 10.1302/0301-620X.69B4.3611151

[b32] Perkins R. & Skirving A. P. Callus formation and the rate of healing of femoral fractures in patients with head injuries. J. Bone Joint Surg. Br. 69, 521–524 (1987). 3611150 10.1302/0301-620X.69B4.3611150

[b33] Smith R. Head injury, fracture healing and callus. J. Bone Joint Surg. Br. 69, 518–520 (1987). 3611149 10.1302/0301-620X.69B4.3611149

[b34] Genet F. . Neurological heterotopic ossification following spinal cord injury is triggered by macrophage-mediated inflammation in muscle. J. Pathol. 236, 229–240 (2015). 25712044 10.1002/path.4519

[b35] Baldock P. A. . Neuropeptide y attenuates stress-induced bone loss through suppression of noradrenaline circuits. J. Bone Miner. Res. 29, 2238–2249 (2014). 24535841 10.1002/jbmr.2205

[b36] Rosen C. J. Bone remodeling, energy metabolism, and the molecular clock. Cell Metab. 7, 7–10 (2008). 18177720 10.1016/j.cmet.2007.12.004

[b37] Swift J. M., Hogan H. A. & Bloomfield S. A. beta-1 adrenergic agonist mitigates unloading-induced bone loss by maintaining formation. Med. Sci. Sports Exerc. 45, 1665–1673 (2013). 23470310 10.1249/MSS.0b013e31828d39bc

[b38] Takeda S. Central control of bone remodeling. Biochem. Biophys. Res. Commun. 328, 697–699 (2005). 15694403 10.1016/j.bbrc.2004.11.071

[b39] Baldock P. A. . Hypothalamic regulation of cortical bone mass: opposing activity of Y2 receptor and leptin pathways. J. Bone Miner. Res. 21, 1600–1607 (2006). 16995815 10.1359/jbmr.060705

[b40] Baldock P. A. . Hypothalamic Y2 receptors regulate bone formation. J. Clin. Invest. 109, 915–921 (2002). 11927618 10.1172/JCI14588 PMC150931

[b41] Gautschi O. P. . Osteoinductive effect of cerebrospinal fluid from brain-injured patients. J. Neurotrauma 24, 154–162 (2007). 17263679 10.1089/neu.2006.0166

[b42] Bidner S. M., Rubins I. M., Desjardins J. V., Zukor D. J. & Goltzman D. Evidence for a humoral mechanism for enhanced osteogenesis after head injury. J. Bone Joint Surg. Am. 72, 1144–1149 (1990). 2398084

